# Management of Congenital Diaphragmatic Hernia (CDH): Role of Molecular Genetics

**DOI:** 10.3390/ijms22126353

**Published:** 2021-06-14

**Authors:** Giulia Cannata, Chiara Caporilli, Federica Grassi, Serafina Perrone, Susanna Esposito

**Affiliations:** 1 Pediatric Clinic, Pietro Barilla Children’s Hospital, University of Parma, Via Gramsci 14, 43126 Parma, Italy; cannata.giulia@gmail.com (G.C.); chiara.caporilli@studenti.unipr.it (C.C.); federica.grassi1@studenti.unipr.it (F.G.); 2 Neonatology Unit, Pietro Barilla Children’s Hospital, Department of Medicine and Surgery, University of Parma, 43126 Parma, Italy; Serafina.perrone@unipr.it

**Keywords:** birth defects, congenital diaphragmatic hernia, diaphragm embryology, genetics, whole exome sequencing

## Abstract

Congenital diaphragmatic hernia (CDH) is a relatively common major life-threatening birth defect that results in significant mortality and morbidity depending primarily on lung hypoplasia, persistent pulmonary hypertension, and cardiac dysfunction. Despite its clinical relevance, CDH multifactorial etiology is still not completely understood. We reviewed current knowledge on normal diaphragm development and summarized genetic mutations and related pathways as well as cellular mechanisms involved in CDH. Our literature analysis showed that the discovery of harmful *de novo* variants in the fetus could constitute an important tool for the medical team during pregnancy, counselling, and childbirth. A better insight into the mechanisms regulating diaphragm development and genetic causes leading to CDH appeared essential to the development of new therapeutic strategies and evidence-based genetic counselling to parents. Integrated sequencing, development, and bioinformatics strategies could direct future functional studies on CDH; could be applied to cohorts and consortia for CDH and other birth defects; and could pave the way for potential therapies by providing molecular targets for drug discovery.

## 1. Introduction

Congenital diaphragmatic hernia (CDH) consists of a life-threatening developmental defect in variable size in the fetal diaphragm that allows abdominal viscera to herniate into the chest [1]. CDH occurs in approximately 1 in 3000 live births [2]. Despite improvements in survival with advanced diagnostic techniques along with medical and surgical care, the average mortality rate worldwide is 50%, depending primarily on pulmonary hypoplasia, pulmonary hypertension, and heart failure [3,4]. Long-term morbidity among survivors is common [5], including longer postnatal hospital stays of affected neonates, poor growth, developmental delay, gastroesophageal reflux, and chronic oxygen dependence [6].

Congenital diaphragmatic defects are variable in size encompassing diaphragm agenesis; well-circumscribed defects or “holes”; and, less often, a thinning or undermuscularization of diaphragmatic tissue. Prenatal ultrasound detection is successful in 50% of CDH cases at a mean gestational age of 24 weeks; fetal magnetic resonance imaging (MRI), fetal echocardiography, and three-dimensional ultrasound imaging also play a role in the study of congenital diaphragmatic hernia, including diagnosis, severity stratification, and prognostic prediction [7,8]. The vast majority of neonates with CDH present with cardiorespiratory distress within the first hours or days of life; however, about 5% to 25% of diaphragmatic hernias present beyond the neonatal period (late-presenting CDH), and clinical manifestations may include respiratory or gastrointestinal symptoms, or a combination of both [9,10,11].

Despite its high impact on neonatal health, pathogenesis and etiology of CDH remain poorly understood. CDH is thought to be multi-factorial, with genetic, environmental, and nutritional factors playing a role [7,12,13]. The involvement of multiple genetic factors is suggested by recent advances in our understanding of the genetic pathways regulating normal diaphragm development and genetic mutations leading to CDH. New insights have been produced after the development and availability of new genetic testing procedures including whole genome sequencing (WGS) and whole exome sequencing (WES) [14,15]. Monogenic syndromic disorders, single-gene mutations, multiple chromosomal abnormalities such as deletions, and aneuploidies have been reported to be associated to CDH [16,17].

This review of the literature provides an overview of current knowledge of normal diaphragm embryogenesis and a summary of genetic mutations as well as related pathways and cellular mechanisms involved in CDH whose knowledge is essential to the development of new therapeutic strategies and evidence-based genetic counselling to parents. Systematic searches were performed in PubMed, Embase, Cochrane Library, Scopus, Google Scholar, and ClinicalTrials.gov accessed on 30 March 2021. Language was restricted to English. Search terms included congenital diaphragmatic hernia (CDH), diaphragm development and embryology, molecular genetic pathways, genetics, and genetic testing. Case reports, case series, original research studies, review articles, letters to the editor, randomized controlled trials (RCTs), non-RCTs, and cohort studies (prospective or retrospective) published were included.

## 2. Diaphragm Development

### 2.1. Diaphragm Basic Anatomy

The diaphragm muscle is a dome-shaped musculotendinous structure [18]. Because of distinct attachment sites, the muscular part of diaphragm is bilaterally divided into sternal, costal, and lumbar parts. Muscular fibers originate from the internal and the external arcuate ligaments, from the lateral lower six ribs on each side and from the sternum. The muscle fibers from these attachments converge in a three-leaf shaped central tendon, forming the crest of the dome. The right lateral leaf is the largest, the left lateral one is the smallest, and the anterior leaf is intermediate in size and is fused with the diaphragmatic surface of the pericardium.

**Figure 1:**
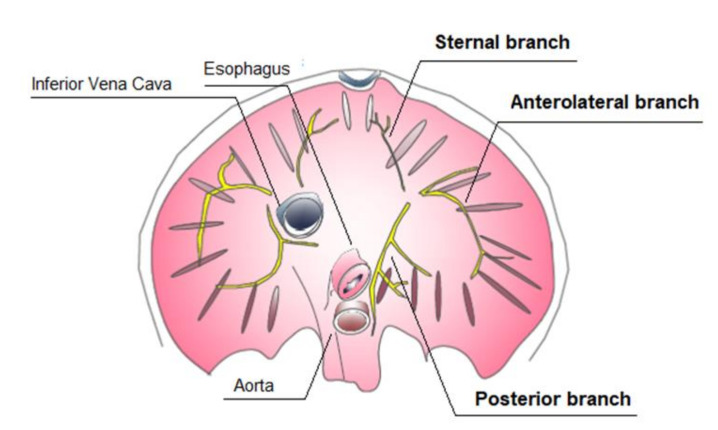
Inferior view of diaphragm: intramuscular distribution of the phrenic nerve.

The diaphragm is a passageway for structures from the thorax to the abdomen: the major orifices are the aortic hiatus, vena caval foramen, and the oesophageal hiatus. The phrenic nerve provides motor and sensory innervation to the diaphragm. The right and left phrenic nerves originate in the spinal cervical roots (C3, C4, and C5), and each phrenic nerve innervates half of the diaphragm. Both nerves always divide into a variable number of macroscopic branches, from two to seven, varying in size and thickness [18]. Three main branches are described: anterior, lateral, and posterior ones. The anterior branches run anteromedially toward the sternum, the anterolateral branches run laterally anterior to the lateral edges of the central tendon, while the posterior branches divide into two rami: posterolateral and posterior [19,20] (Figure 1).

The presence of an accessory phrenic nerve arising from the fifth or fifth and sixth cervical nerves has been noted [21].

### 2.2. Anatomic Findings

**Figure 2:**
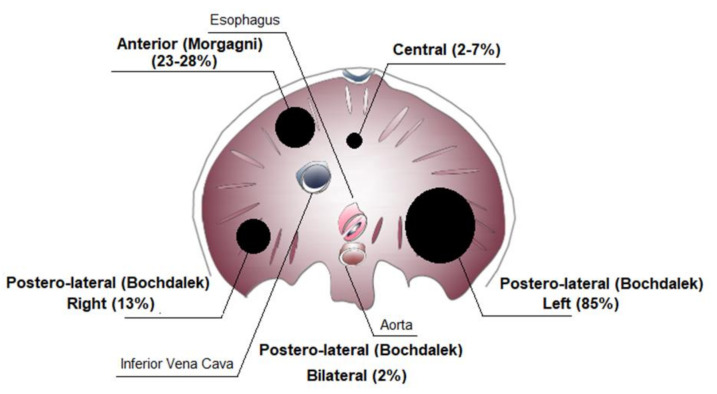
Congenital diaphragmatic hernia (CDH) classification.

CDH is classified according to the location of the defect in the diaphragm (Figure 2). Posterolateral diaphragmatic hernias, referred to as Bochdalek hernias, are the most common hernia type (70–75%), with the majority occurring on the left side (85%), and less frequently on the right side (13%) or bilaterally (2%). Anterior defects also known as Morgagni–Larrey hernias (23–28%) and central hernias (2–7%) are the other types [1,22,23].

The size of the defect can range from small to the most extreme variant consisting of complete agenesis [24].

### 2.3. Diaphragm Embryogenesis

**Figure 3:**
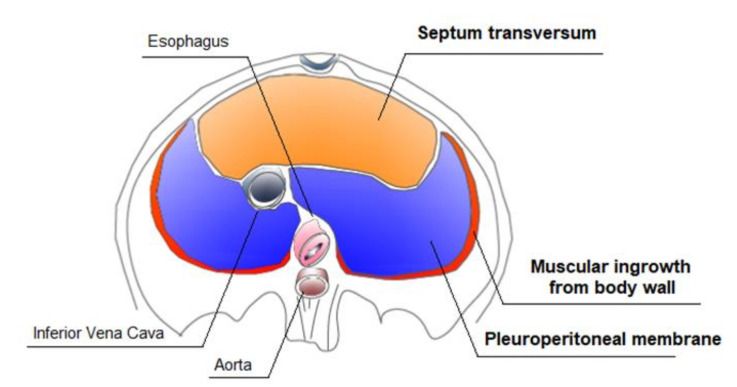
Development of the diaphragm.

Defects in diaphragm development lead to CDH. Recently, progress has been made in understanding the genetic pathways regulating diaphragm development and the genetic mutations leading to CDH. Some CDH-associated genetic mutations and related defects in different molecular pathways can directly affect diaphragm development and of other organs, such as the lungs and the hearth [25,26]. Transgenic mouse models’ studies have helped to understand normal diaphragm development [27]. The mature diaphragm derives from multiple embryonic sources: the septum transversum, the pleuroperitoneal folds (PPFs), muscular ingrowths from body wall, and the dorsal mesentery of esophagus (Figure 3).

The largest contributors are the pleuroperitoneal folds (PPFs), two transient pyramidal structures appearing at the beginning of the fifth week of pregnancy and situated between the thoracic and abdominal cavities. The PPFs expand dorsally and ventrally, merging with the septum transversum and mesentery of the esophagus, giving rise to the diaphragm’s muscle connective tissue and central tendon in the seventh week of pregnancy, definitively separating the thoracic cavity from the from the abdominal cavity. An additional rim originating from the body wall forms the most peripheral part of the diaphragm [27]. The muscle progenitors migrate from cervical somites to the developing diaphragm into the PPFs [28,29]. After reaching the pleuroperitoneal folds, the muscle progenitors undergo the myogenic program controlled by the network of myogenic regulatory transcription factors (MRF), including Myf5, MyoD, and myogenin [30,31]. Defects in pre-muscular cell migration, differentiation, and proliferation lead to the formation of an abnormal diaphragm musculature [32].

Similar to the muscle progenitors, the phrenic nerve axons originating from the cervical segments of the neural tube reach the PPFs via a ventromedial pathway. Subsequently, the phrenic nerve splits into three branches and elongates in close association with the dorsal and central expansion of the muscle [29,33]. Correct primary and secondary phrenic nerve branching and motoneuron axon targeting through the formation of neuromuscular junctions with differentiated myofibers during development are critical for diaphragmatic function [34,35].

PPF-derived signals regulate migration of muscle and neural progenitors to the developing diaphragm: outgrowth of the phrenic nerve may be guided by neural cell adhesion molecule (NCAM) and low-affinity nerve growth factor receptor (NGFR) expressed along the path from the neural tube to the pleuroperitoneal folds. In addition, hepatocyte growth factor (HGF), the ligand for the Met receptor, is expressed along the migratory path for both the muscle progenitors and the nerves [33,36].

## 3. Prognostic Factors

### 3.1. Prenatal Prognostic Factors

CDH is a pathology diagnosed in prenatal age [37]. Prenatal diagnosis of CDH is possible as early as 12 weeks of gestation during first trimester ultrasound screen; CDH ultrasound detection is successful in 50% of cases at a mean gestational age of 24 weeks. Fetal magnetic resonance imaging (MRI), three-dimensional ultrasound imaging, and fetal echocardiography also play a role in prenatal CDH detection and severity stratification [8,22,38]. The main determinants of CDH outcomes are the presence of associated abnormalities, particularly heart disease, extent of pulmonary hypoplasia, and liver position (intra-abdominal or intrathoracic) [8].

The prognosis of isolated CDH is better than CDH associated with multiple abnormalities; a higher survival rate has also been demonstrated for the former [39,40]. Liver position is a survival index: liver herniation (liver up) is associated with a worse prognosis. Metkus et al. reported a survival rate of 100% in CDH cases without hepatic hernia (liver-down) as compared to 56% with liver herniation (liver-down) [41]. Mullassery et al. have described a decrease in survival from 73.7% to 45.4% with liver herniation; liver herniation as a marker for survival showed a sensitivity of 73%, a specificity of 54%, a positive predictive value of 54%, and a negative predictive value of 73% [42].

Prenatal prognostic indicators also include gestational age at diagnosis [43,44], stomach position [45,46,47], polyhydramnios [43], lung size [41], mediastinal shift [48], lung-to-head ratio (LHR) [41,49,50,51], and the preferable observed to expected normal mean for gestation lung-to-head ratio (O/E LHR) [52,53,54,55]. Survival is less than 50% with an O/E LHR of less than 25% and exceeds 80% with an O/E LHR greater than 40%.

As regards stomach position in prediction of survival, Basta et al. established degrees of herniation on ultrasound examination: grade 1, stomach not visualized; grade 2, stomach visualized anteriorly, next to apex of heart, with no structure in between stomach and sternum; grade 3, stomach visualized along from apex of heart and abdominal structures anteriorly; or grade 4, which is the same as grade 3 with stomach posterior to level of atrioventricular heart valves [46].

Lastly, additional indexes derived from imaging modalities other than two-dimensional ultrasound, such as three-dimensional ultrasound, Doppler ultrasonographic analysis of fetal pulmonary vasculature, fetal echocardiography, and magnetic resonance, have been found to be risk predictors of pulmonary hypertension, extra corporeal membrane oxygenation (ECMO) need, and prognostic factors for survival [8,56,57].

### 3.2. Postnatal Prognostic Factors

Very often, prenatal prognostic factors are insufficient to predict postnatal risk. Many neonates are born without a prenatal diagnosis. Prenatal imaging can often be limited. It appears that the birth weight, Apgar score at 5 min > 7, the severity of respiratory failure (measured by the highest values of fraction of inspired oxygen (FiO_2_), mean arterial pressure (MAP), Oxygenation Index (OI), and alveolar–arterial oxygen gradient (AaDO_2_)), and primary pulmonary hypertension (PPH) act as independent factors in influencing the survival rate of patients with CDH [58]. Congenital Diaphragmatic Hernia Study Group (CDHSG) predicted survival, CDHSG defect size, Willford Hall/Santa Rosa clinical prediction formula, Brindle score, and SNAP-II are validated postnatal prediction tools. CDHSG predictive survival is applied in the immediate postnatal period and is based on the Apgar score at 5 min and birth weight to generate a probability of survival [51]. CDHSG defect size is based on the size of the defect that is a marker significantly correlated with survival, need for patch repair, and other adverse outcomes [59,60]. The Willford Hall/Santa Rosa clinical prediction formula uses the difference between the highest value of partial pressure of arterial oxygen (PaO_2_) and the highest value of partial pressure of carbon dioxide (PCO_2_) in the first 24 h of life to generate a score of survival [51]. The variables of the Brindle score are instead very low birth weight, absent or low Apgar score at the fifth minute, echocardiographic evidence of pulmonary hypertension, or cardiac or chromosomal abnormalities, and are defined as a mortality score [61]. SNAP-II is a survival score calculated in the first 24–48 h of life on the basis of mean blood pressure, lowest temperature, PO_2_/FiO_2_ ratio, lowest serum pH, seizure activity, and urine output [51,62].

Persistent pulmonary hypertension and pulmonary hypoplasia are believed to be responsible for the high morbidity [63,64]. The degree of pulmonary hypoplasia is almost impossible to assess before and immediately after birth, and in most cases, the degree of pulmonary hypoplasia is an important determining factor in the outcome. In addition, pulmonary hypertension (PHT) causes an increase in the right ventricle (RV) after loading, progressively compromising the RV function. Dysfunction and dilation of the RV, in turn, lead to left ventricular (LV) dysfunction, potentially contributing to adverse clinical outcomes [7,65]. Aggarwal et al. in fact demonstrated that LV dysfunction was associated with death and negative outcomes, justifying incorporation of echocardiographic indices as prognostic markers of CDH [66].

Neonates with left-sided CDH had a significantly lower left ventricular mass assessed by echocardiography than ones with other causes of persistent pulmonary hypertension of the newborn (PPHN). The reduced left ventricular mass contributes to functional LV hypoplasia and may result in increased left atrial pressure, pulmonary venous hypertension, and a reduced left ventricular output [67,68]. Yamoto et al. revealed an association of pulmonary circulation parameters predictive of poor CDH prognosis, including the following echocardiographic parameters: right-to-left (R-L) shunt, right pulmonary artery (RPA) diameter, left pulmonary artery (LPA) diameter and left ventricular dimension at diastole (LVDd) between patients who survived up to 90 days and those who died. Smaller RPA diameter and LVDd were good predictors of mortality in CDH [69].

## 4. Classification of Congenital Diaphragmatic Hernia (CDH)

### Isolated and Non-Isolated Congenital Diaphragmatic Hernia (CDH)

**Figure 4:**
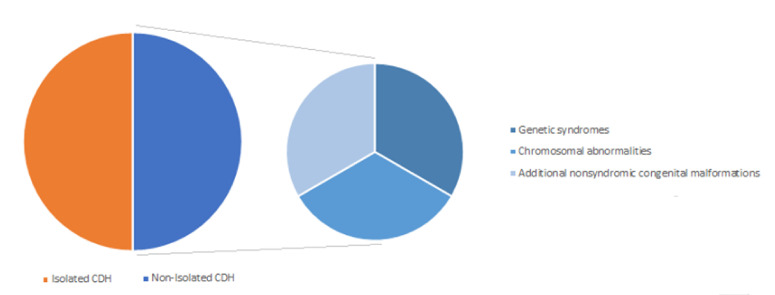
Classification of congenital diaphragmatic hernia (CDH).

CDH hernia may occur in isolation (isolated CDH) in approximately 50% of cases or in association with additional congenital anomalies for the remainder (non-isolated CDH or CDH +), either as part of a genetic syndrome, a chromosome abnormality, or a nonsyndromic collection of major congenital malformations (Figure 4) [16].

Pulmonary hypoplasia, intestinal malrotation, and left hearth hypoplasia may coexist with isolated CDH and are usually considered part of a sequence. Nonsyndromic major congenital malformations associated with non-isolated CDH may involve the cardiovascular (27.5%), urogenital (17.7%), musculoskeletal (15.7%), and the central nervous system (9.8%) [70].

Many patients with CDH are the only family members affected, and therefore this condition has been typically interpreted as sporadic. The majority of CDH cases are sporadic and appear to be multifactorial. The etiology of most cases remains unknown; however, there is increasing evidence that genetic factors may play a role in the development of CDH.

Genetic contributions to CDH are heterogeneous, identified from animal models, conventional karyotype, chromosome microarray, and next-generation sequencing technology, including whole exome sequencing (WES) and whole genome sequencing (WGS) [71].

Chromosomal aberrations have been discovered as an important etiology for CDH, reported in about 10% of CDH cases and detected by routine karyotyping and chromosome microarray. Complete and mosaic aneuploidies, copy number variant (CNV) deletions, insertions, duplications, and translocations are included in this percentage. Nearly all chromosomes may be affected by structural anomalies [72,73,74,75].

## 5. Genetic Contribution to Congenital Diaphragmatic Hernia (CDH)

### 5.1. Aneuploidies

Aneuploidy is a major chromosomal anomaly in which chromosome number is abnormal.

Different aneuploid conditions are associated with CDH: trisomy 18, trisomy 13, trisomy 21, and Turner syndrome (45, X) are the most frequent aneuploidies reported in association.

#### 5.1.1. Trisomy 21—Down Syndrome

Congenital Morgagni’s hernia (CMH), also called congenital Morgagni–Larrey’s hernia, is rare when compared with other types of congenital diaphragmatic hernia, accounting for about 23–28% of all types of CDH [1,22,23].

Congenital Morgagni’s hernia (CMH) can be associated with trisomy 21 with a variable reported incidence ranging from 14% to 50% [76,77,78,79].

The age of presentation varies from the neonatal period to adulthood [80,81,82,83].

#### 5.1.2. Trisomy 18—Edwards Syndrome

Trisomy 18 is the most prevalent aneuploidy associated with CDH cases. CDH occurs more often in male fetuses than in trisomy 18 female fetuses [84].

#### 5.1.3. Trisomy 13—Patau Syndrome

Association of CDH with trisomy 13 (Patau syndrome) has been rarely described [85,86].

#### 5.1.4. Turner syndrome (45, X) and Trisomy X (46, XXX)

Turner syndrome (45, X) and Trisomy X (46, XXX) have been rarely described in association with CDH [87,88].

#### 5.1.5. Tetrasomy 12p—Mosaic Isochromosome 12p Syndrome—Pallister–Killian Syndrome (OMIM 601803)

Pallister–Killian syndrome is caused by mosaicism for tetrasomy of chromosome 12p [89,90,91]. It is characterized by seizures; mental retardation; skin abnormalities; and coarse facial features, including prominent forehead, hypertelorism, short nose, anteverted nostrils, and flat occiput [92,93,94]. A high rate of association between Pallister–Killian syndrome and congenital diaphragmatic hernia has been described in the literature [95,96].

### 5.2. Copy Number Variants (CNVs)

#### 5.2.1. Deletion 1q41-q42 (OMIM 612530)

Clinical features of patients diagnosed with deletion of 1q41-q42 region include dysmorphic features (deep-set eyes, frontal bossing, broad nasal tip, anteverted nares, and depressed nasal bridge), microcephaly, seizures, and neurodevelopmental disabilities, as well as, less commonly, cleft palate, clubfeet, and congenital diaphragmatic hernia [97,98]. Deletion in this region involves the candidate genes H2.0-like homeobox (*HLX*) and dispatched RND transporter family member 1 (*DISP1*) [99,100,101,102].

#### 5.2.2. Duplication 1q25q31.2

Few CDH cases associated with duplication of 1q25q31.2 have been reported in literature [103].

#### 5.2.3. Deletion 3q22

Deletion in this region involves the candidate genes for cellular retinol binding protein 1 (*RBP1*) and cellular retinol binding protein 2 (*RBP2*), which play a role in retinol signaling pathway, essential for embryonic development [104,105]. CDH cases associated with this deletion have been reported in the literature [106,107].

#### 5.2.4. Deletion 4p16 (OMIM 194190)

Deletion of chromosomal region 4p16, also known as Wolf–Hirschhorn syndrome, is characterized by pre- and postnatal growth retardation; microcephaly; “Greek helmet” facies; mental retardation; seizures; and/or epilepsy and closure defects including cleft lip or palate, cardiac septal defects, and coloboma of the eye [108,109,110]. Although not a common finding, CDH has been described in association with Wolf–Hirschhorn syndrome [111,112,113,114].

#### 5.2.5. Deletion 6p25

CDH has been described in individuals with 6p25 deletion [115].

#### 5.2.6. Deletion 8q23 (OMIM 610187)

Few CDH cases associated with deletion on chromosome 8q23 have been reported in the literature. This chromosomal region harbors zinc finger protein and FOG family member 2 (*ZFPM*2, FOG2) genes [116]. *Fog2* is required for normal diaphragm and lung development in mice and humans [25]. Variants and deletions in *ZFMP2* have been described in patients with CDH [117].

#### 5.2.7. Deletion 8p23 (OMIM 222400)

Deletions of 8p23.1 of variable size have been reported in association with complex congenital heart anomalies, CDH, facial dysmorphisms, and neurodevelopmental disabilities [118,119,120,121,122,123]. Deletion in this region involves the candidate gene *GATA4*, encoding GATA4 zinc finger DNA-binding protein involved in heart, lung, and diaphragm development [26,124].

#### 5.2.8. Duplication of 8p21-p23.1

Duplication of 8p21-p23.1 has been described in association with CDH [125,126,127].

#### 5.2.9. Deletion 9p24-pter

Terminal deletions of this region have been described in patients with nonisolated CDH [128,129].

#### 5.2.10. Deletion 11p13

Deletion of chromosomal region 11p13, harboring the Wilms tumor 1 gene (*WT1*), has been described in association with CDH cases [130,131]. *WT1* plays a crucial role in diaphragm development [132].

#### 5.2.11. Duplication 11q23.3-qter

Duplication of 11q23.3-qter has been reported in several cases of CDH [133,134].

#### 5.2.12. Deletion 15q26

Non-isolated CDH cases have been described in association with deletion of the distal long arm of chromosome 15 [135]. *NR2F2*, also known as chick ovalbumin upstream promoter-transcription factor II (*COUP-TFII*), harbored in this chromosomal region, is believed to play a role in retinoic acid metabolism and diaphragm development [135,136].

### 5.3. Monogenic Syndromes

Well-characterized monogenic syndromes have been associated with CDH, accounting for approximately 3–10% of all CDH cases [74,137]. Associated syndromes include both X-linked and autosomal-dominant and -recessive inheritance [138]. No one genetic cause accounts for more than 1–2% of CDH cases. Below, we summarize the most known monogenic syndrome causes of CDH. Both de novo and inherited variants contribute to CDH [138].

#### 5.3.1. Simpson–Golabi–Behmel Syndrome (OMIM 312870)

Simpson–Golabi–Behmel syndrome (SBGS) is a X-linked recessive disorder due to mutations in glypican-3 (*GPC3*) [139]. It is characterized by prenatal and postnatal macrosomia; coarse facial features; hypertelorism; macroglossia; macrocephaly; skeletal; cardiac and renal abnormalities; thoracoabdominal wall defects; and, commonly, mild to severe intellectual disability. CDH has been reported in 24% of individuals with Simpson–Golabi–Behmel syndrome, accounting for about 34% of all types of gastrointestinal and abdominal wall malformations described in association with this syndrome [140,141].

#### 5.3.2. Craniofrontonasal Syndrome (OMIM 304110)

Craniofrontonasal syndrome (CFNS) is an X-linked dominant disorder caused by a variant in Ephrin B1 (*efnb1*). It is characterized by craniosynostosis, hypertelorism, broad nasal tip, grooved nails of the hallux and thumb, syndactyly, and skeletal abnormalities [142]. Male patients show a milder phenotype than females [143]. CDH has been described in both genders [142,143,144].

#### 5.3.3. Myotubular Myopathy 1 (OMIM 310400)

Myotubular myopathy 1 (MTM1) is an X-linked recessive centronuclear myopathy due to loss of function mutations in the myotubularin 1 (MTM1) at Xq28 [145]. Diaphragm eventration and diaphragm ventilator-dependent dysfunction have been described [146].

#### 5.3.4. Opitz G/BBB Syndrome (OMIM 300000)

Opitz G/BBB syndrome is characterized by facial anomalies, genitourinary abnormalities, and laryngotracheoesophageal defects caused by loss of function of *MID1* gene [147]. CDH has been described in association with Opitz G/BBB syndrome [148].

#### 5.3.5. Lowe Syndrome (OMIM 309000)

Lowe syndrome, also called oculocerebrorenal syndrome, is a multisystemic disorder characterized by the triad of proximal renal tubular dysfunction, congenital cataracts, and intellectual disability. It is caused by variants in inositol polyphosphate 5-phosphatase *ocrl-1* [149,150]. CDH has been described in association with this syndrome [151].

#### 5.3.6. Focal Dermal Hypoplasia—Goltz Syndrome (OMIM 305600)

Focal dermal hypoplasia or Goltz syndrome is caused by variants in porcupine O-acyltransferase (*PORCN*) gene [152]. It is characterized by developmental skin malformations and digital, ocular, and dental abnormalities [153,154]. CDH has been reported in in association with Goltz syndrome [155,156].

#### 5.3.7. MIDAS Syndrome (OMIM 309801)

MIDAS syndrome is characterized by unilateral or bilateral microphthalmia and linear areas of aplastic skin limited to the face and neck [157]. Additional features, including CDH, have been described in association with this syndrome [158].

#### 5.3.8. Cornelia de Lange Syndrome (OMIM 122470)

Cornelia de Lange syndrome, also called Brachmann de Lange syndrome, is caused by heterozygous variants in *NIBL* gene at chromosome 5p13.1, encoding for the NIPBL protein [159]. Distinctive clinical features are prenatal and postnatal growth restriction, facial dysmorphisms, developmental delay, malformations of the upper extremities, heart defects, and gastrointestinal and genitourinary malformations [160]. CDH is a recognized clinical feature of Cornelia de Lange syndrome [161,162,163]. In one large review of 426 patients diagnosed with Cornelia de Lange syndrome, CDH has been identified as the cause of death in 10% of cases [164].

#### 5.3.9. Denys–Drash Syndrome (OMIM 194080)

Denys–Drash syndrome is caused by variants in the tumor-suppressor gene *wt1*. It is characterized by male pseudohermaphroditism, nephrotic syndrome leading to end-stage renal disease, and increased risk of development of Wilms’ tumor [165]. To date, only three CDH cases associated with Denys–Drash syndrome have been reported in literature [166,167,168]. *WT1* plays a crucial role in diaphragm morphogenesis as confirmed by the development of congenital diaphragmatic hernia in *wt1*-null mouse embryos. However, the mechanisms regulated by *WT1* are unknown [132].

#### 5.3.10. Marfan Syndrome (OMIM 154700)

Marfan syndrome is a heritable disorder of fibrous connective tissue with a great clinical variability. It is a multisystemic disease with ocular, skeletal, cardiovascular, and pulmonary involvement. Marfan syndrome results from mutations in the *fbn1* gene encoding fibrillin-1, an extracellular matrix protein [169,170]. To date, over 3000 *FBN1* gene variants have been identified [171].

Diaphragmatic abnormalities are rare features in Marfan syndrome. Severe neonatal presentations have been described [172,173], and approximately 20% of patients with early onset Marfan syndrome have a diaphragmatic eventration [174].

#### 5.3.11. CHARGE Syndrome (OMIM 214800)

CHARGE syndrome results from variants within the chromodomain helicase DNA-binding protein 7 gene (*CHD7*) located on 8q12 [175,176]. CHARGE is an acronym for coloboma, heart defects, choanal atresia, retardation (growth and/or development retardation), genitourinary malformation, and ear abnormalities [177,178]. The diagnosis is based on major and minor criteria, as published by Blake et al. and modified by Verloes [179,180]. CDH has been rarely described in CHARGE syndrome [181,182].

#### 5.3.12. Fryns Syndrome (OMIM 229850)

Fryns syndrome has been reported to be the most common autosomal recessive syndrome associated with CDH, accounting for 1.3%–10% of all CDH cases [137,183,184,185]. Recessive variants in the phosphatidyl inositol glycan biosynthesis type N (*PIGN*) gene are the genetic basis of disease [186,187].

Diagnostic criteria for Fryns syndrome include diaphragmatic defect, characteristic facial appearance, distal digital hypoplasia, pulmonary hypoplasia, at least one characteristic associated anomaly, and family history consistent with autosomal recessive inheritance [188]. The syndrome encompasses a broad spectrum of diaphragmatic developmental defects including diaphragmatic hernia in any location, eventration, agenesis or significant hypoplasia of the diaphragm [189,190].

#### 5.3.13. Donnai–Barrow Syndrome (OMIM 222448)

Donnai–Barrow syndrome is a rare autosomal recessive entity characterized by typical facial dysmorphism (hypertelorism, bulging eyes, down slanting palpebral fissures, posteriorly rotated ears, widow’s peak), large anterior fontanel, high-grade myopia, low molecular weight proteinuria, sensorineural deafness, corpus callosum anomalies, and congenital diaphragmatic hernia [191,192].

The syndrome results from variants in the low-density lipoprotein receptor-related protein 2 gene (*LRP2*) encoding megalin, a multi-ligand transmembrane endocytic receptor critical for reuptake of numerous ligands, including lipoproteins, sterols, vitamin-binding proteins, and hormones [193,194]. Megalin has a role in cell signaling by interacting with vitamin A (retinol), critical for diaphragm development [195]. Donnai–Barrow syndrome is associated with CDH in >50% of patients [196,197].

#### 5.3.14. Matthew–Wood Syndrome (OMIM 601186, 615524)

Matthew–Wood syndrome, also known as Spear syndrome or microphthalmic syndrome 9, is caused by homozygous variants in *stra6* (stimulated by retinoic acid 6) gene encoding for a multitransmembrane domain protein acting as retinol-binding protein (RBP) receptor. RBP is the principal physiological carrier of retinol (vitamin A) [198]. RBP receptor binds to RBP and mediates vitamin A uptake from vitamin A-loaded RBP (holo-RBP) [199]. From this, it follows the importance of *stra6* expression and the role of vitamin A during embryonic development.

Matthew–Wood disease is a multisystemic syndrome characterized by pulmonary hypoplasia/aplasia, cardiac malformations, anophthalmia, and diaphragmatic hernia/eventration [200,201,202].

#### 5.3.15. Multiple Vertebral Segmentation Defects

CDH has been reported in association with disorders of vertebral segmentation, variously called Jarcho–Levin syndrome, spondylothoracic dysostosis, and spondylocostal dysostosis [203,204,205,206]. Multiple contiguous vertebral abnormalities (such as hemivertebrae, block vertebrae, or unsegmented bars), rib anomalies (such as absent or fused ribs), and shortened trunk are common features of these disorders [207].

Exact distinction among these disorders is missing. Multiple vertebral segmentation defects can be inherited in an autosomal-dominant or an autosomal-recessive pattern; to date, variants have been described as being related to delta-like 3 (*DLL3*), lunatic fringe (*LFNG*), and mesoderm posterior 2 (*MESP2*) in the Notch signaling pathway, playing a role in somite formation [208]. *DLL3* is the most commonly mutated, inherited in an autosomal recessive pattern [209].

### 5.4. Most Frequent Genes identified by Next-Generation Sequencing and Related Pathways

The text below and Table 1 shows an overview of the most frequent candidate genes and signaling pathways for CDH.

#### 5.4.1. GATA4

GATA4 gene is localized on chromosomal region 8p23.1 and codes for GATA4 zinc finger DNA-binding protein. *GATA4* is a component of the retinoic pathway required for normal diaphragm, heart, and lung development, as confirmed by mutant mice lacking *GATA4* suffering from defective morphogenesis [26,124,210,211]. Chromosomal deletions of 8p23.1 of variable size have been described in association with congenital diaphragmatic hernia [118,119,120,121,122,123].

De novo *GATA4* variants identified by WES have also been described in association with sporadic and familial cases of CDH [212].

#### 5.4.2. GATA6

GATA6 is a zinc finger transcription factor that plays a role in visceral endoderm differentiation and organogenesis [213,214]. GATA6 is essential for pulmonary, diaphragm, and pericardium development [215,216,217,218,219,220]. GATA6 is expressed in murine primordial septum transversum mesenchyme and lateral PPFs [15,221]. Early embryonic lethality has been observed in mice nullizygous for *Gata6* [222].

De novo and inherited variants in *GATA6* have been identified in CDH patients. Yu et al. used WES and identified de novo variants in *GATA6* in patients with CDH and congenital heart disease [223].

#### 5.4.3. FOG2 (ZFPM2)

*FOG2*, also known as *ZFPM2*, gene is located on chromosomal region 8q23 and codes for zinc finger protein ZFPM2, FOG family member 2, which modulates the activity of GATA transcription factors. ZFPM2 protein interacts primarily with *gata4*, which in turn modulates embryonic development. *FOG2* is necessary for both diaphragm and lung development, as underscored in mice nullizygous for *Fog2* gene affected with diaphragmatic eventration and pulmonary hypoplasia [25].

Inherited and de novo variants in *ZFPM2* gene have been described in association with familial and sporadic CDH cases [116,117,224].

#### 5.4.4. COUP-TFII (NRF2)

Chick ovalbumin upstream promoter-transcription factor II (COUP-TFII), also known as NRF2 (nuclear receptor subfamily 2, group F, member 2), has been mapped to chromosome 15q26 and is believed to play a role in retinoic acid metabolism and diaphragm development [135,136,225,226].

*COUP-TFII* has also been associated with other congenital anomalies, including pancreatic agenesis and congenital heart disease [227].

*COUP-TFII* is expressed in the developing diaphragm, in PPFs, and in the transverse septum. *COUP-TFII* is also expressed in the developing lung mesenchyme, suggesting a possible etiological link with lung hypoplasia frequently associated with diaphragmatic defects [226,228].

High et al. have identified by WES that mutations in the coding region of *COUP-TFII*, alone or in combination with other contributing factors, could be a rare cause of CDH. Larger deletions are associated with complex phenotypes, smaller deletions with isolated CDH, or CDH with congenital heart anomalies [227].

#### 5.4.5. SIN3A

SIN3A is a corepressor regulating genes transcription through interaction with retinoic acid receptors (RARs) [229].

*SIN3A* gene mutations have been described in human CDH cases. *SIN3A* is required for normal lung and diaphragm development, as confirmed by *Sin3a* mutant mice [230].

#### 5.4.6. MYRF (MRF)

Myelin regulatory factor (MYRF), also known as myelin gene regulatory factor (MRF), is a membrane-associated transcription factor that is highly expressed in developing heart and diaphragm. *MYRF* mutations have been described in association with congenital heart defects (hypoplastic left heart syndrome, scimitar syndrome, septal defects, and valvular anomalies), genitourinary anomalies (ambiguous genitalia, hypospadias, and cryptorchidism), congenital diaphragmatic hernia, and pulmonary hypoplasia [231,232].

## 6. Implications of Congenital Diaphragmatic Hernia (CDH) Diagnosis

CDH can occur as an isolated defect or complex defect associated with other congenital anomalies. Chromosomal abnormalities, affecting the entire chromosome or partial aneuploidies, are the most common genetic causes recognized for CDH [16,233,234,235,236]. Karyotype, chromosomal microarray, and WES are the techniques used in the prenatal period capable of identifying about 30% of the causes of non-isolated CDH [74,237]. Overall, 10% of chromosomal abnormalities causative for CDH are identified by chromosomal microarray analysis [74]. WES/WGS represents the best method to assess the contribution of de novo mutations.

The purpose of identifying the cause is to discover and clarify the risk of recurrence. It is essentially a careful family history in the identification of the cause of CDH. Trio studies (proband, mother, and father) have been carried out to identify de novo and recessive mutations, and their importance as tools for identifying undiagnosed genetic conditions is emerging. Serious undiagnosed developmental disorders are based on de novo harmful mutations in developmentally important genes [238]. Most patients with CDH have no family history of CDH, leading to the hypothesis that de novo variants are an important etiological mechanism [239].

However, WES and WGS should be reserved to particular cases because of the high costs and difficult to identify new candidate genes. Approximately 10–22% of CDH isolated and complex patients have a de novo sequence variant that can be identified through the use of WES/WGS [237,240,241,242]. Recent advances in WES and WGS have had an advantage in facilitating detection of de novo (as well as those inherited) single-nucleotide variants (SNVs), short insertions and deletions (indels), and copy number variations (CNVs). This has significant implications for genetic counseling for parents and risk stratification of recurrence. These techniques also represent a useful tool for the diagnosis of new syndromes but also for the counseling and follow-up of newborns born with apparently isolated congenital defects.

## 7. Conclusions

CDH is a relatively common major life-threatening birth defect that results in significant mortality and morbidity depending primarily on lung hypoplasia, persistent pulmonary hypertension, and cardiac dysfunction. Despite its clinical relevance, CDH multifactorial etiology is still not completely understood. Genetic contributions to CDH appeared heterogeneous. Advances in genomics, coupled with functional studies in animal models, are increasingly identifying the causes of CDH in both familial and sporadic cases [243,244,245]. Through these approaches, we are beginning to elucidate the mechanisms and molecular pathways that are responsible for diaphragm and lung development abnormalities in CDH patients. A key challenge will be to understand which molecular pathways are most commonly disrupted and contribute to diaphragm and lung defects in CDH. An additional challenge will be to understand what causes the phenotypic variability and different clinical outcomes of CDH patients who share the same genetic mutation. A better insight into the mechanisms regulating diaphragm development and genetic causes leading to CDH appeared essential to the development of a personalized approach, new therapeutic strategies, and evidence-based genetic counselling to parents. The discovery of harmful de novo variants in the fetus could constitute an important tool for the medical team (gynecologists, neonatologists, geneticists, anesthetists, and pediatric surgeons) during pregnancy. Integrated sequencing, development, and bioinformatics strategies could direct future functional studies on CDH, could be applied to cohorts and consortia for CDH and other birth defects, and could pave the way for potential therapies by providing molecular targets for drug discovery. Due to genetic predispositions for CDH, there is the possibility of recurrence in later pregnancies [246]. This information should be shared with parents to help them make an informed choice between expectant management and prenatal referral for elective delivery; termination of pregnancy; or, in selected patients, fetal intervention. Presence of a human element in medical communication increases parents’ confidence in healthcare professionals and enhances their emotional resilience and preparedness [246].

## Figures and Tables

**Table 1:** Overview of candidate genes and signaling pathways for congenital diaphragmatic hernia (CDH).

Gene	Gene Locus	Protein Activity	Expression	Function	Anomalies Associated
*COUP-TFII*	15q26.2	Nuclear receptor subfamily 2 group F: steroid/thyroid hormone receptor superfamily	Lung mesenchyme	Transcription factor	CHD, pancreatic agenesis
*SIN3A*	15q24.2	Corepressor that interacts with the retinoic acid receptor (RARs)		Transcription factor	Microcephaly, intellectual disabilities
*FOG2*	8q23.1	Zinc finger protein that modulates the transcriptional activity of GATA proteins	Lung mesenchyme and diaphragm	Transcription cofactor	Cardiac defects, lung hypoplasia, eventration
*GATA4*	8q23.1	Zinc finger transcription factor; retinoic signaling pathway	Liver, lung mesenchyme, diaphragm	Transcription factor	Cardiac defects, eventration of the diaphragm
*GATA6*	18q11.1-q11.2	Zinc finger containing transcription factors	Lung epithelium and diaphragm	Transcription factor	Organogenesis defects of the pancreas and heart and defect in the development of the diaphragm and pericardium
*MYRF*	11q12.2	Membrane-associated transcription factor	Diaphragm, lung, heart, genitourinary system	Transcription factor	CHD, genitourinary anomalies, and pulmonary hypoplasia

CHD, congenital heart disease.

## Data Availability

Not applicable in a review article.
